# BDNF val66met association with serotonin transporter binding in healthy humans

**DOI:** 10.1038/tp.2016.295

**Published:** 2017-02-14

**Authors:** P M Fisher, B Ozenne, C Svarer, D Adamsen, S Lehel, W F C Baaré, P S Jensen, G M Knudsen

**Affiliations:** 1 Neurobiology Research Unit, Copenhagen University Hospital Rigshospitalet, Copenhagen O, Denmark; 2 Department of Public Health, Section of Biostatistics, University of Copenhagen, Copenhagen K, Denmark; 3 PET and Cyclotron Unit, Copenhagen University Hospital Rigshospitalet, Copenhagen O, Denmark; 4 Danish Research Centre for Magnetic Resonance, Centre for Functional and Diagnostic Imaging and Research, Copenhagen University Hospital Hvidovre, Hvidovre, Denmark

## Abstract

The serotonin transporter (5-HTT) is a key feature of the serotonin system, which is involved in behavior, cognition and personality and implicated in neuropsychiatric illnesses including depression. The brain-derived neurotrophic factor (BDNF) val66met and 5-HTTLPR polymorphisms have predicted differences in 5-HTT levels in humans but with equivocal results, possibly due to limited sample sizes. Within the current study we evaluated these genetic predictors of 5-HTT binding with [^11^C]DASB positron emission tomography (PET) in a comparatively large cohort of 144 healthy individuals. We used a latent variable model to determine genetic effects on a latent variable (5-HTT_LV_), reflecting shared correlation across regional 5-HTT binding (amygdala, caudate, hippocampus, midbrain, neocortex, putamen and thalamus). Our data supported a significant BDNF val66met effect on 5-HTT_LV_ such that met-carriers showed 2–7% higher subcortical 5-HTT binding compared with val/val individuals (*P*=0.042). Our data did not support a BDNF val66met effect in neocortex and 5-HTTLPR did not significantly predict 5-HTT_LV_. We did not observe evidence for an interaction between genotypes. Our findings indicate that met-carriers have increased subcortical 5-HTT binding. The small difference suggests limited statistical power may explain previously reported null effects. Our finding adds to emerging evidence that BDNF val66met contributes to differences in the human brain serotonin system, informing how variability in the 5-HTT level emerges and may represent an important molecular mediator of BDNF val66met effects on behavior and related risk for neuropsychiatric illness.

## Introduction

Serotonin is an important neurotransmitter implicated in many aspects of behavior and cognition.^1^ Dysfunction of the brain serotonin system is thought to play a role in neuropsychiatric illnesses including depressive and anxiety disorders.^2^, ^3^ Therefore, identifying sources of variability in serotonin signaling may help elucidate mechanisms that drive variability in behavior and related risk for illness. Brain-derived neurotrophic factor (BDNF) is a signaling molecule that critically affects synaptic plasticity, axonal growth and cell survival.^4^ Furthermore, compelling evidence from animal models and human studies indicate that BDNF affects the serotonin system.^5^, ^6^ Within the human *BDNF* gene a common single-nucleotide polymorphism (BDNF val66met, rs6265) has been identified wherein the met-allele is associated with reduced activity-dependent BDNF release.^7^ This polymorphism has been associated with inter-individual variability in multiple behavioral phenotypes including memory performance, aggression and fear recall.^7^, ^8^, ^9^, ^10^ The met-allele has been associated with increased vulnerability for depression in the presence of stressful life events.^11^ Another recent study reported an over-representation of met/met individuals with post-traumatic stress disorder and exaggerated startle responses,^12^ consistent with an association between the met-allele and impaired fear extinction.^9^ Therefore, identifying serotonin mechanisms sensitive to BDNF val66met genotype would elucidate its role as a potential molecular mediator of this variant on behavior and risk for neuropsychiatric illness.

Molecular neuroimaging genetics studies using positron emission tomography (PET) have reported that compared with BDNF val66met val/val individuals, met-carriers show decreased serotonin 1A receptor (5-HT_1A_) binding,^13^ but see also Henningsson *et al.*,^14^ and increased serotonin 4 receptor (5-HT_4_) binding, a putative marker for brain serotonin levels^15^ but no differences in serotonin 2A receptor binding.^16^ The BDNF val66met effect on serotonin transporter (5-HTT) binding is unclear as previous studies have reported lower binding in met-carriers but only in males^14^ and no genotype differences.^16^, ^17^ The 5-HTT is a key component of the serotonin system as it is the central mechanism by which serotonin is cleared from the extracellular space.^18^ However, the relatively small sample size of these studies (*N*=25–52) coupled with the infrequency of the met-allele (~0.2 in European populations) suggests these studies are limited in identifying a potentially small BDNF val66met effect. Furthermore, BDNF val66met effects may have been moderated by additional genetic variants such as the commonly studied 5-HTTLPR polymorphism in the 5-HTT gene (*SLC6A4*), which in some studies has been shown to affect 5-HTT binding,^19^, ^20^, ^21^ but see also Parsey *et al.*^22^and Murthy *et al.*^23^ and moderate effects of BDNF val66met on brain function^24^ and personality.^25^ Here we aimed to further characterize the effect of BDNF val66met on 5-HTT levels in a comparatively large cohort of 144 healthy individuals using [^11^C]DASB PET.

## Materials and methods

### Participants

Data were included from the Cimbi database.^26^ All participants were recruited by advertisement for different research protocols approved by the Ethics Committee of Copenhagen and Frederiksberg, Denmark ((KF) 01-124/04, (KF) 01-156/04, (KF) 01 2006-20, H-1-2010-085, H-1-2010-91, H-2-2010-108, including amendments). Written informed consent was obtained from all participants after a complete description of the respective study. Although inclusion criteria varied slightly across studies, (for example, some studies recruited only males or females), all participants included here were generally healthy and without: 1) primary psychiatric disease, 2) substance or drug abuse and 3) severe systemic or neurological disease based on self-reported history and physical/neurological examination. Additional criteria for inclusion in this study were: (1) a [^11^C]DASB PET scan before any intervention, (2) available BDNF val66met and 5-HTTLPR genotype, (3) self-identified European ancestry and (4)⩽51 years old (to limit partial volume effects and because of a clear break in the age distribution of [^11^C]DASB scans between 51 and 63 years of age). [^11^C]DASB PET scans were acquired between 2005 and 2012. Subgroups of individuals with PET data presented here have been included in previous studies.^27^, ^28^, ^29^, ^30^, ^31^, ^32^, ^33^, ^34^ Most notably, data presented here were included in previous reports of BDNF val66met^16^ and 5-HTTLPR effects on 5-HTT binding.^19^

### Genotyping

BDNF val66met genotype was determined as previously described.^15^ 5-HTTLPR genotype, including the A/G single-nucleotide polymorphism (rs25531), was determined as previously described.^35^ BDNF val66met genotype was in Hardy–Weinberg equilibrium (*χ*^2^=1.6, (degree of freedom) df=1, *P*=0.20), whereas this test is not appropriate for 5-HTTLPR because this was an inclusion criterion for some studies.^32^, ^33^

### MRI data acquisition

A high-resolution T1-weighted structural brain scan was acquired for each participant and used for segmentation and delineation of regions of interest. Scans were acquired on one of two magnetic resonance imaging (MRI) scanners, a Siemens Magnetom Trio 3 T scanner or a Siemens Verio 3 T scanner (Siemens, Erlangen, Germany).

### [^11^C]DASB PET data acquisition

PET scans were acquired on one of two PET scanners, an 18-ring GE-Advance scanner (General Electric, Milwaukee, WI, USA) operating in three-dimensional (3D)-acquisition mode with an approximate in-plane resolution of 6 mm or a Siemens ECAT high-resolution research tomograph (HRRT) scanner operating in 3D-acquisition mode with an approximate in-plane resolution of 2 mm. Following a 10- or 6-min transmission scan (Advance and HRRT, respectively), an intravenous bolus injection of [^11^C]DASB was given over 12 or 20 s (Advance and HRRT scanner) and a dynamic 90-min emission scan was acquired over 36 frames (6 × 10 s, 3 × 20 s, 6 × 30 s, 5 × 60 s, 5 × 120 s, 8 × 300 s, 3 × 600 s). Dynamic PET images acquired on the Advance scanner were reconstructed using filtered back projection and corrected for attenuation, dead-time and scatter using a 6-mm Hann filter. Dynamic PET images acquired on the HRRT scanner were reconstructed using an iterative OP-OSEM3D method with resolution modeling (10 iterations and 16 subsets).^36^, ^37^, ^38^

The automatic image registration algorithm was used to determine single-subject within PET scan motion and realignment.^39^ PET scans were smoothed using a 12- or 10-mm within-frame Gaussian filter before alignment (Advance and HRRT). We estimated rigid translation/rotation parameters aligning each PET frame to a single PET frame with sufficient structural information using the scaled least squares cost function (frame 26: 20–25 min post injection). Non-filtered PET images were resliced using these parameters. Co-registration of high-resolution MR and PET images was performed using automatic image registration or SPM (Advance and HRRT) based on the mean of frames 10–26, corresponding to a flow-weighted image. Accurate co-registration was confirmed by visual inspection across all planes.

Regions were automatically delineated on the participant's structural MRI scan using Pvelab.^40^ Time-activity curves reflecting the mean of gray-matter voxels within each region were determined except for the midbrain region of interest where the mean time-activity curve across all voxels was used. Regional binding potential estimates (BP_ND_) were determined from kinetic modeling of regional time-activity curves in PMOD (Zurich, Switzerland) using the multilinear reference tissue model (MRTM/MRTM2) with a fixed k2' estimated for each individual using a striatal high binding region and cerebellum as the reference region.^41^ Bilateral regional BP_ND_ values were calculated by computing a volume-weighted mean from the left and the right hemisphere.

### Data analysis

Statistical analyses were carried out in R v3.2.2 (https://cran.r-project.org/). The primary outcome measures were regional BP_ND_ values for neocortex (including occipital-, orbitofrontal-, parietal–cortex, pre/post central-, middle/inferior frontal-, middle/inferior temporal-, superior frontal- and superior temporal-gyrus), amygdala, caudate, hippocampus, midbrain and thalamus. A single neocortex region was selected in part because of previous observations of particularly high correlation between cortical regions.^27^ Genotype groupings for all analyses were BDNF val66met: val/val versus met-carriers (at least one met-allele) and 5-HTTLPR: L_A_L_A_ vs S'-carriers (at least one L_G_ or S-allele). Age, sex, PET scanner, MRI scanner, weight-adjusted injected mass of DASB, daylight minutes on day of PET scan and body-mass index were considered as covariates and retained as supported by the data. All continuous variables were mean-centered and dichtomous variables were dummy coded as 0/1.

Latent variable models were estimated using the *lava* package^30^ in R. We modeled all regional BP_ND_ values onto a single latent variable (5-HTT_LV_), reflecting shared correlation in BP_ND_ across regions.^27^ We then attempted to model all covariate effects through 5-HTT_LV_. PET and MRI scanner effects were modeled directly on to each regional BP_ND_ estimate because of evidence for region-specific effects, consistent with our previous observation with a different radioligand.^15^ The addition of model paths was considered iteratively, based on Wald tests of improvement in model fit with a false-discovery rate of *q*<0.05 (Benjamini–Hochberg false-discovery rate-corrected) across all possible paths. This allowed us to effectively determine the inclusion of model paths supported by the data while controlling the rate of false-positive model paths. Overall, model fit was assessed by comparison of our model against a saturated model, root mean square error of approximation (RMSEA) and support for additional model paths.^42^ Regional BP_ND_ residuals were visually inspected for normality. An identifiable model was chosen such that covariate effects reported can be interpreted in terms of effects on thalamus BP_ND_ (reference scale). The entire model was estimated simultaneously and *P*-values<0.05 (two-sided) were considered statistically significant.

Corresponding univariate linear regression models were estimated for each region. Reported percent differences in 5-HTT BP_ND_ reflect the BDNF val66met parameter estimate expressed as a fraction of the intercept parameter estimate for the corresponding linear regression model, multiplied by 100. Unless otherwise stated, results are reported with parameter estimates and 95% confidence intervals in brackets with associated units.

### Code availability

R code for latent variable model or other analyses described in the manuscript can be made available upon request.

## Results

Demographic and PET scan information is detailed in Table 1. Notably, genotype groups were well balanced across variables and there was no association between BDNF val66met and 5-HTTLPR genotype (*χ*^2^=0.02, df=1, *P*=0.90).

An initial evaluation of regional BP_ND_ indicated that they were highly intercorrelated (Supplementary Figure 1). Consistent with this, loadings on the single latent variable, 5-HTT_LV_, strongly supported shared correlation across regions (all loadings: *P*<10^−^^12^). Weight-adjusted injected DASB mass, daylight minutes and body mass index were not significantly associated with 5-HTT_LV_ and removed from the model. Additional shared inter-regional correlations between (1) caudate and putamen, (2) midbrain and thalamus and (3) amygdala and hippocampus were supported by Wald tests (*q*<0.03). Wald tests supported an additional direct effect of BDNF val66met on neocortex (*q*=0.02). Sex was not predictive of 5-HTT_LV_ but Wald tests supported a direct effect on caudate (*q*=0.02). Subsequently, no additional paths were supported by Wald tests (*q*>0.3), consistent with good overall model fit. Fit of our final model was less than that of a saturated model (log-likehood ratio test: *X*^2^=50.9, df=34, *P*=0.03), suggesting less than ideal model fit. However, RMSEA was within commonly accepted bounds (RMSEA=0.058, 90% confidence interval (CI): (0.018, 0.09)) and no additional model paths were supported (*q*>0.3), suggesting good model fit. Adding the most strongly (but not significantly) supported additional path more closely aligned model fit with the saturated model (log-likelihood ratio test: *P*=0.07) without substantively affecting genotype effects. Taken together, we retained the model excluding this not significantly supported path to limit the addition of false-positive paths and conclude good but not ideal model fit.

**Figure 1:**
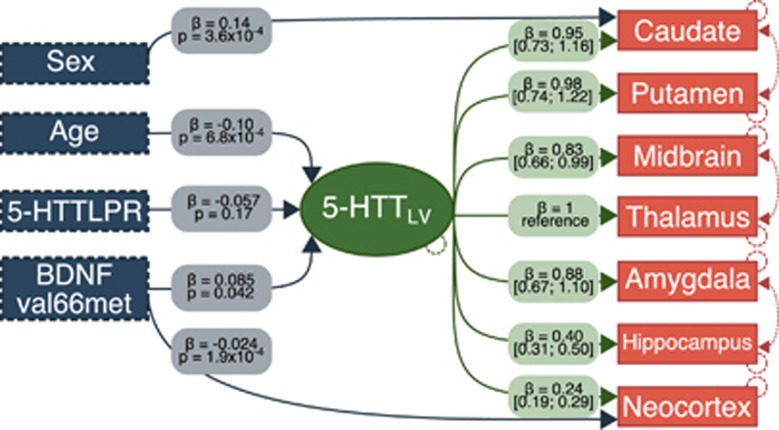
Latent variable model. Blue hatched boxes represent observed predictors. The green oval represents the estimate latent variable, 5-HTT_LV_. Orange solid boxes represent measured regional 5-HTT BP_ND_ values. Lines denote included model paths. Although not indicated, MRI scanner and PET scanner are modeled as predictors of regional 5-HTT BP_ND_. BDNF val66met val/val and 5-HTTLPR L_A_L_A_ are reference groups for respective parameter estimates. Hatched lines between regions indicate additional shared correlation. Hatched circles indicate error estimates included. Parameter estimates, *β*, for each model path indicated in respective boxes with either 95% confidence interval or *P*-value noted. All regions significantly loaded on the latent variable (all factor loadings: *P*<10^−12^). 5-HTT, serotonin transporter; BDNF, brain-derived neurotrophic factor; MRI, magnetic resonance imaging; PET, positron emission tomography.

**Figure 2:**
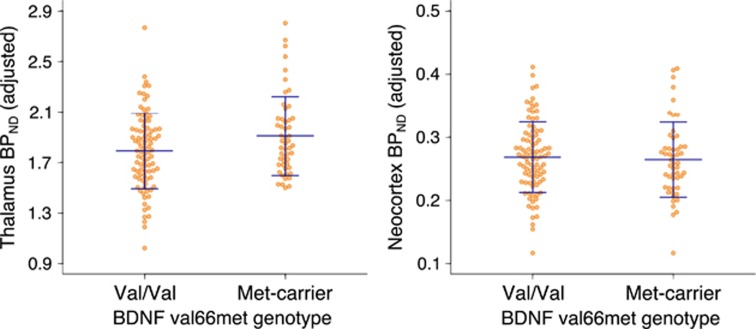
Representative BDNF val66met effects on thalamus and neocortex 5-HTT BP_ND_. BDNF val66met met-carriers showed higher 5-HTT BP_ND_ in thalamus and other subcortical regions but not neocortex (see Table 2 for model estimates). Orange dots represent 144 individual 5-HTT BP_ND_ values, adjusted for age, MRI scanner and PET scanner. Blue lines represent group mean±1 s.d. 5-HTT, serotonin transporter; BDNF, brain-derived neurotrophic factor; MRI, magnetic resonance imaging; PET, positron emission tomography.

Our final model is depicted in Figure 1. Genetic effects in corresponding univariate models can be found in Table 2. Within our final model BDNF val66met significantly predicted 5-HTT_LV_ (met-carriers versus val/val: 0.085 (0.0031, 0.17), *P*=0.042, units: thalamus BP_ND_), which significantly captured shared correlation in 5-HTT BP_ND_ across regions. More specifically, met-carriers showed 2–7% higher 5-HTT BP_ND_ across subcortical brain areas compared with val/val individuals (Figure 2). The BDNF val66met effect on neocortex BP_ND_ is the sum of the direct (BDNF val66met → neocortex BP_ND_) and indirect (BDNF val66met → 5-HTT_LV_ → neocortex BP_ND_) effects, which indicates no genotype effect on this region (total met-carrier versus val/val neocortex effect: −0.004, (−0.024, 0.016), units: neocortex BP_ND_; Figure 2). We also observed a significant negative effect of age on 5-HTT_LV_ (estimate: −0.098 (−0.15, −0.042), *P*=0.00069, units: thalamus BP_ND_ per decade) and a caudate-specific effect of sex (male versus female: 0.14 (0.062, 0.21), *P*=0.00036, units: caudate BP_ND_). MRI and PET scanner effects are reported in Supplementary Table 1. We did not find evidence for an interaction between 5-HTTLPR and BDNF val66met in predicting 5-HTT_LV_ (*P*=0.11).

## Discussion

Here we evaluated BDNF val66met effects on 5-HTT binding within a comparatively large molecular neuroimaging data set of 144 healthy individuals using a latent variable model framework. Our findings support that 5-HTT binding across the brain is well described by a single latent variable (5-HTT_LV_), reflecting significant shared correlation across regions. BDNF val66met significantly predicted subcortical 5-HTT binding as evidenced by an effect on 5-HTT_LV_ but no effect on neocortex 5-HTT binding. Met-carriers showed 2–7% higher subcortical 5-HTT binding compared with val/val individuals. 5-HTTLPR did not significantly predict 5-HTT binding in our model nor did we observe evidence for an interaction between genotypes. Our significant but relatively small group differences may explain equivocal findings from previous studies. This finding provides additional evidence for an effect of BDNF val66met on the adult human brain serotonin system, which may represent a molecular mediator of its effects on behavior, personality and risk for neuropsychiatric illness.

Previous reports of BDNF val66met effects on 5-HTT binding have been inconsistent where one study reported decreased 5-HTT binding in met-carriers, but only in 16 males from a cohort of 25 individuals.^14^ Our group and another previously reported nonsignificant effects on 5-HTT binding in 52 and 41 individuals, respectively.^16^, ^17^ Here we report significantly greater 5-HTT binding in met-carriers across subcortical regions. Considering the relatively small difference in 5-HTT binding (2–7%) that we observed, it is quite plausible that previous studies were underpowered to detect an effect. Notably, we observed a significant effect of BDNF val66met including 43 of the data sets from our previous study (nine excluded because of age).^16^ Considering evidence that the BDNF val66met met-allele is associated with reduced trafficking and activity-dependent BDNF release,^7^ our findings indicate these effects contribute to an increase in 5-HTT levels as indexed by [^11^C]DASB PET. Linking genetic variation with differences in neurobiology enables our ability to more comprehensively model neurobiology via easily acquired genetic information. Our current findings provide a more complete representation of how BDNF val66met shapes individual differences in serotonin signaling, which is implicated in myriad aspects of behavior, brain function and neuropsychiatric illnesses.

We did not find evidence for a BDNF val66met effect on neocortex 5-HTT binding. This is reflected by the sum of the direct and indirect paths between these two variables. The data-driven addition of the direct BDNF val66met → neocortex path in our model significantly improved model fit, indicating that the BDNF effect on neocortex via the latent variable insufficiently captures its effect on neocortex. We previously observed evidence for a neocortex-specific effect of 5-HTTLPR on 5-HT_4_ binding in healthy controls.^15^ Considering that 5-HTT BP_ND_ is relatively low in neocortex, our null finding may reflect a type-II error stemming from low signal-to-noise. Alternatively, evidence from non-human primates and rodent models indicates that serotonin projections to cortex are sparse relative to subcortical projections and evidence relatively few synapses, suggesting volume transmission.^43^, ^44^, ^45^ As such, it is also possible that 5-HTT levels in neocortex are regulated differently compared with subcortical regions, affecting sensitivity to genetic variation. Regardless, our findings suggest that, although there is high correlation in 5-HTT BP_ND_ across regions, suggesting the presence of ‘global regulators', region-specific effects should also be considered.

We did not find evidence for a 5-HTTLPR effect on 5-HTT_LV_, although nominally decreased 5-HTT_LV_ in S'-carriers is consistent with *in vitro* evidence of reduced *SLC6A4* transcription.^46^ Similar previous molecular neuroimaging genetics studies are mixed with some reporting reduced 5-HTT binding in S-carriers,^19^, ^20^, ^21^ whereas other studies have reported no effect.^22^, ^23^ Epigenetic information was not available for these data but should be considered in future studies as epigenetic factors such as DNA methylation in the *SLC6A4* gene could have moderated 5-HTTLPR effects on 5-HTT binding, possibly explaining the limited correspondence between *in vitro* and *in vivo* 5-HTTLPR effects on 5-HTT levels. Here, S'-carriers showed particularly lower 5-HTT BP_ND_ in the amygdala, a central brain region for processing salient environmental stimuli and previously linked both to 5-HTTLPR genotype and 5-HTT BP_ND_ (Table 2).^47^, ^48^, ^49^ Thus, although it is an intriguing possibility that 5-HTT levels mediate a 5-HTTLPR effect on amygdala function and related behavioral phenotypes, our current findings further suggest this effect is limited in size and therefore would require a uniquely large multimodal neuroimaging sample.

Unsurprisingly, BP_ND_ estimates were consistently higher on the HRRT compared with Advance scanner. The higher-resolution HRRT limits spill-in effects of neighboring areas with low binding. However, it is notable that the magnitude of this effect varied across regions, which we could model within this structural equation model framework, highlighting its strength in studies pooling heterogeneous data. We also observed evidence for a difference in 5-HTT BP_ND_ between MRI scanners such that midbrain 5-HTT BP_ND_ was higher in Verio scans. Notably, only women were scanned on this scanner, who showed nominally higher midbrain BP_ND_ compared with the men considering only Trio scans in a univariate framework (male versus female midbrain BP_ND_, Trio scans only: −0.08 (−0.22, 0.056)), which may have contributed to this difference. The resolution and quality of T1-weighted high-resolution Verio and Trio scanners are comparable, and we are therefore cautious in interpreting this observation but encourage future studies to consider potential MRI-related effects where relevant.

We observed a negative effect of age on 5-HTT_LV_, consistent with other PET markers of the serotonin system,^50^, ^51^, ^52^ corresponding to a 2–10% decrease in regional 5-HTT BP_ND_ per decade. In addition, caudate 5-HTT BP_ND_ only was significantly higher in males compared to females. We previously reported this region-specific effect considering only the Advance PET data included in the current study.^19^, ^30^ Remarkably, we observed a similar effect considering only the HRRT PET data (male versus female caudate BP_ND_, HRRT scans only: 0.27 (0.028, 0.52), see Supplementary Table 2 for other regional estimates), providing additional evidence for our previously reported region-specific effect of sex on caudate 5-HTT BP_ND_ in an independent cohort.

Two recent studies have reported BDNF val66met effects on 5-HT_4_ and 5-HT_1A_ binding in healthy adults.^13^, ^15^ It is not clear whether BDNF val66met effects stem from related or parallel genetic effects but these receptors show notably distinct neuronal distributions. The 5-HTT is primarily expressed on serotonin neurons, whereas 5-HT_1A_ is located on non-serotonin neurons (except in raphe) and 5-HT_4_ is primarily expressed in striatum on interneurons and projection medium spiny neurons.^53^, ^54^ It is also not known whether BDNF val66met-induced differences emerge during development; however, this is plausible, given the prominent role in development of both systems and evidence that BDNF manipulation during development affects the serotonin system.^55^, ^56^, ^57^, ^58^ Although our findings diverge from a study in mice that reported heterozygous knockout of *Bdnf* decreased 5-HTT levels in hippocampus,^59^ we are cautious in directly comparing such findings to single-nucleotide polymorphism effects in humans because of similar divergent findings with other BDNF–serotonin associations.^16^, ^60^ Further, the net BDNF val66met effect on the serotonin system is likely best described by its effect on a broader serotonin system feature set, rather than only 5-HTT binding. As such, multitracer PET studies wherein 5-HTT, 5-HT_4_, 5-HT_1A_ binding and other serotonin measures are acquired within the same individuals would provide a more complete estimate of serotonin system features, enabling a more comprehensive assessment of how serotonin signaling is regulated by BDNF val66met and other genetic or epigenetic mechanisms.

Our study is not without its limitations. Our cohort includes data pooled across studies with varying inclusion criteria. Nevertheless, all participants met a common basic criteria of general health, all data were collected and processed in the same manner, including the same quality-control checks throughout processing. Another source of heterogeneity is regionally variable scanner differences. However, BDNF val66met effects were similar when considering only HRRT scans (BDNF val66met effect on 5-HTT_LV_, HRRT scans only: 0.11, (0.0035, 0.22)), suggesting that our effect is not confounded by scanner effects. Although we conclude a BDNF val66met effect on 5-HTT_LV_ and not each region independently, this can be viewed as a sensible trade-off, given compelling evidence for shared correlation across regions (Supplementary Figure 1). This indicates that the alternative, a univariate test for each region, fails to leverage shared information across regions. Consistent with this notion, we would not find evidence for a statistically significant BDNF val66met effect on any region with a univariate strategy following reasonable multiple comparison correction (Table 2). This emphasizes the value of modeling shared correlation in multivariate frameworks such as latent variable models, which is highly relevant to molecular neuroimaging where regional binding measures are often intercorrelated and effect sizes may be small.^27^

In summary, our findings indicate that BDNF val66met met-carriers show significantly increased subcortical 5-HTT binding (2–7% across regions) but no effect in neocortex. We replicate a negative correlation with age and evidence that males show higher BP_ND_ in caudate. These findings provide additional evidence for BDNF val66met effects on the brain serotonin system, a potential molecular mediator of its effects on behavior, personality and related risk for neuropsychiatric illness.

## Figures and Tables

**Table 1:** Demographic and [^11^C]DASB PET scan information

	*Total*	*BDNF val66met*		*5-HTTLPR*	
		*val/val*	*met-carrier*	*L*_*A*_*L*_*A*_	*S'-carrier*
N	144	92	52 (9 met/met)	42	102 (53 S'S')
Age (mean±s.d.)	26.7±7.1	26.4±7.2	27.0±7.1	25.9±5.4	27.0±7.7
Sex (F/M)	85/59	54/38	31/21	24/18	61/41
Body mass index	23.6±2.9	23.3±2.7	24.3±3.1	24.0±3.0	23.5±2.9
Daylight minutes	669±219	671±219	666±220	632±201	685±225
PET scanner (A/H)	43/101	31/61	12/40	13/29	30/72
MRI scanner (T/V)	84/60	53/39	31/21	22/20	62/40
k2' (per min)	0.066±0.013	0.065±0.013	0.066±0.012	0.066±0.014	0.065±0.012
AUC_cerebellum_ (Bq ml^−1^)	16494±4531	16413±4418	16 638±4763	16 271±4426	16 586±4591
[^11^C]DASB-injected mass (μg)	3.16±3.09	3.18±2.91	3.13±3.43	2.85±2.93	3.29±3.16
[^11^C]DASB-injected dose (MBq)	552±78	553±72	550±87	562±73	548±80

Abbreviations: μg, microgram; A, GE-Advance PET scanner; AUC, area under the curve for reference region [^11^C]DASB time-activity curve; BDNF, brain-derived neurotrophic factor; F, female; M, male; MBq, mega-bequerel; MRI, magnetic resonance imaging; H, HRRT PET scanner; PET, positron emission tomography; k2', [^11^C]DASB kinetic modeling parameter; T, Trio MRI scanner; V, verio MRI scanner.

No significant group differences for either genotype, for any measure (*P*>0.05).

**Table 2:** Genetic effects on regional [^11^C]DASB binding from univariate models

*Region*	*BDNF val66met*				*5-HTTLPR*			
	*Estimate*	*95% CI*	P	P_*FWE*_	*Estimate*	*95% CI*	P	P_*FWE*_
Amygdala	0.036	−0.060, 0.13	0.46	1.00	−0.102	−0.20, −0.0012	0.05	0.33
Caudate	0.102	0.0046, 0.20	0.04	0.24	−0.090	−0.19, 0.012	0.08	0.50
Hippocampus	0.016	−0.027, 0.059	0.47	1.00	−0.018	−0.063, 0.028	0.44	1.00
Midbrain	0.086	−0.0034, 0.18	0.06	0.29	0.003	−0.091, 0.097	0.95	1.00
Neocortex	−0.004	−0.024, 0.016	0.70	1.00	−0.015	−0.036, 0.0066	0.18	0.88
Putamen	0.106	−0.0040, 0.22	0.06	0.29	−0.043	−0.16, 0.073	0.46	1.00
Thalamus	0.118	0.011, 0.23	0.03	0.21	−0.009	−0.12, 0.10	0.87	1.00

Abbreviations: BDNF, brain-derived neurotrophic factor; CI, confidence interval; FWE, family-wise error; MRI, magnetic resonance imaging; PET, positron emission tomography.

Effects are expressed as minor allele (met-carrier or S-carrier) relative to major allele (val/val or LL). Age, PET scanner, MRI scanner and sex (caudate only) included as covariates in regression models. *P*_FWE_ reflects Bonferroni–Holm correction across seven regions.
