# Supplementary material for: BDNF val66met association with serotonin transporter binding in healthy humans
Source: Transl Psychiatry. 2017 Feb 14;7(2):e1029–. doi: 10.1038/tp.2016.295 (PMC5438027; doi:10.1038/tp.2016.295)
Supplement: Supplementary Information [file tp2016295x1.docx]

BDNF val66met association with serotonin transporter binding in healthy humans

Supplementary Information

Patrick MacDonald Fisher, Ph.D.^1*^, Brice Ozenne, Ph.D.^1,2^, Claus Svarer, Ph.D.^1^, Dea Adamsen, Ph.D.^1^, Szabolcs Lehel, Ph.D.^3^, William Frans Christian Baaré, Ph.D.^4^, Peter Steen Jensen, M.S.^1^, Gitte Moos Knudsen, M.D., Ph.D.^1^

^1^Neurobiology Research Unit, Copenhagen University Hospital Rigshospitalet, 2100 Copenhagen O, DENMARK; ^2^Department of Public Health, Section of Biostatistics, University of Copenhagen, 1014 Copenhagen K, DENMARK; ^3^PET and Cyclotron Unit, Copenhagen University Hospital Rigshospitalet, 2100 Copenhagen O, DENMARK; ^4^Danish Research Centre for Magnetic Resonance, Centre for Functional and Diagnostic Imaging and Research, Copenhagen University Hospital Hvidovre, 2650 Hvidovre, DENMARK

Summary of two supplementary files:

1. Fisher_etal_BDNF-DASB_SI – This document contains the figure legend for the supplementary figure and two supplementary tables.
2. Fisher_etal_BDNF-DASB_SuppFig1.eps – Supplementary figure file

Supplementary Figure Legend

Supplementary Figure 1. 5-HTT BP_ND_ correlation matrix across regions for all 144 individuals. Ovals represent correlation coefficient (rho) between any two regions. Narrower and redder circles indicate higher correlation. Color bar indicates correlation coefficients.

| Supplementary Table 1. Scanner effects and variance explained by 5-HTT_LV_ | | | | | | |  |
| --- | --- | --- | --- | --- | --- | --- | --- |
|  | **MRI scanner** | | | **PET scanner** | | | **Variance explained by 5-HTT_LV_** |
| *Region* | *Estimate* | *95% CI* | *p* | *Estimate* | *95% CI* | *p* |  |
| Amygdala | 0.047 | [-0.065, 0.16] | 0.41 | 0.276 | [0.16, 0.39] | 4.02x 10^-6^ | 0.40 |
| Caudate | 0.014 | [-0.12, 0.14] | 0.83 | 0.502 | [0.38, 0.62] | 6.28x 10^-17^ | 0.35 |
| Hippocampus | -0.011 | [-0.060, 0.038] | 0.66 | 0.068 | [0.016, 0.12] | 0.010 | 0.52 |
| Midbrain | 0.120 | [0.017, 0.22] | 0.02 | 0.126 | [0.016, 0.24] | 0.024 | 0.43 |
| Neocortex | -0.017 | [-0.041, 0.0068] | 0.16 | 0.174 | [0.15, 0.20] | 2.85x 10^-42^ | 0.32 |
| Putamen | -0.061 | [-0.19, 0.065] | 0.34 | 0.625 | [0.49, 0.76] | 2.32 x 10^-20^ | 0.28 |
| Thalamus | 0.115 | [-0.011, 0.24] | 0.07 | 0.804 | [0.67, 0.94] | 1.70 x 10^-32^ | 0.19 |
| Effects are expressed as Verio compared to Trio and HRRT compared to Advance scanner effects. | | | | | | |  |

| Supplementary Table 2. Regional effects of sex on [^11^C]DASB binding, HRRT scans only | | | |
| --- | --- | --- | --- |
| *Region* | *Estimate* | *95% CI* | *p* |
| Amygdala | -0.007 | [-0.25, 0.23] | 0.95 |
| Caudate | 0.274 | [0.028, 0.52] | 0.03 |
| Hippocampus | 0.012 | [-0.092, 0.12] | 0.82 |
| Midbrain | 0.035 | [-0.17, 0.24] | 0.74 |
| Neocortex | -0.011 | [-0.061, 0.038] | 0.65 |
| Putamen | 0.225 | [-0.051, 0.50] | 0.11 |
| Thalamus | 0.095 | [-0.16, 0.35] | 0.46 |
| Effects are expressed as males relative to females. BDNF val66met, 5-HTTLPR, age, PET scanner and MRI scanner included as covariates in regression models. P-values reported are uncorrected. | | | |
