# Supplementary figures and images for: BDNF val66met association with serotonin transporter binding in healthy humans
Source: Transl Psychiatry. 2017 Feb 14;7(2):e1029–. doi: 10.1038/tp.2016.295 (PMC5438027; doi:10.1038/tp.2016.295)

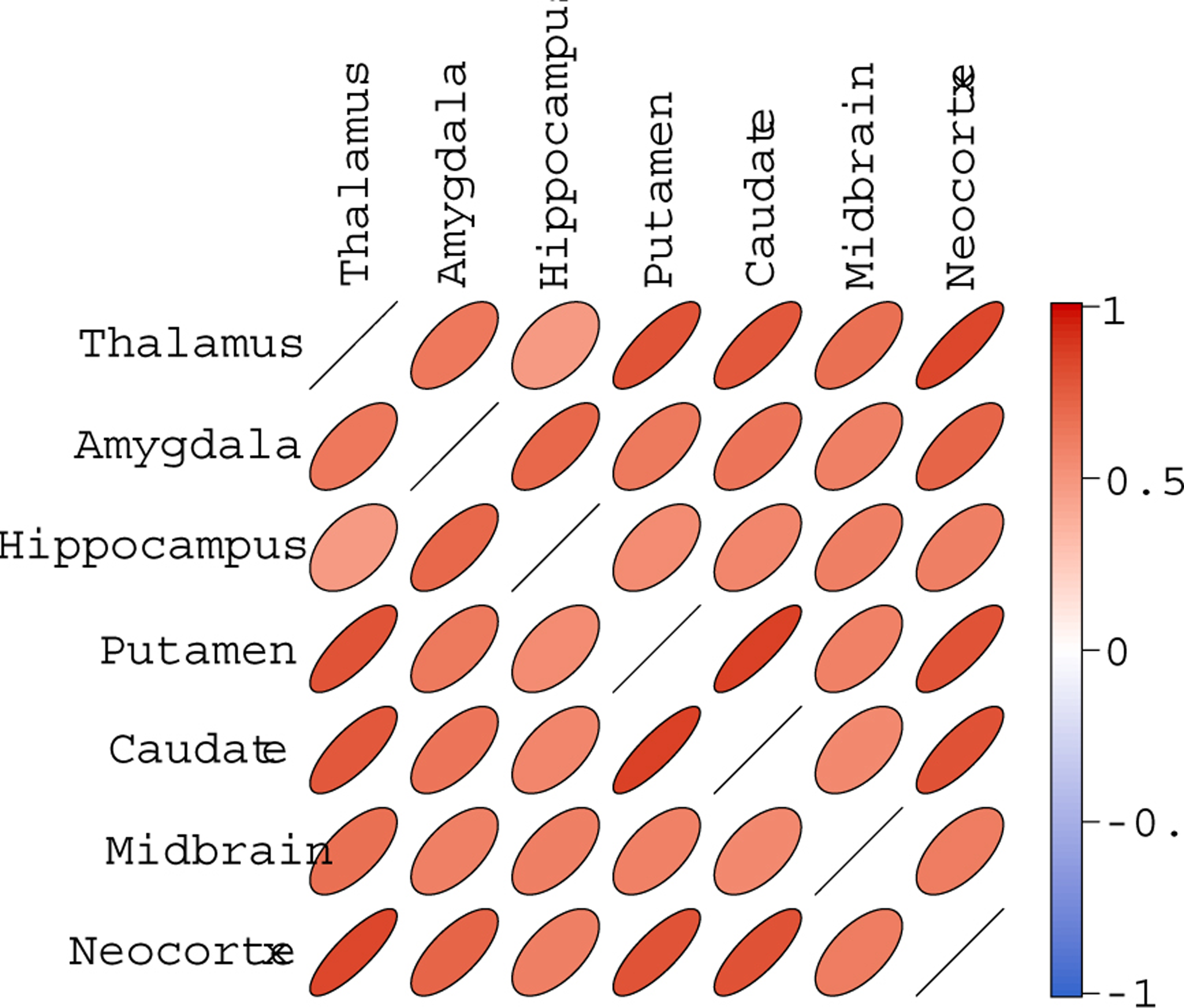

Supplement: Supplementary Figure 1 [file tp2016295x2.tif]
